# In Vivo Bone Effects of a Novel Bisphosphonate‐EP4a Conjugate Drug (C3) for Reversing Osteoporotic Bone Loss in an Ovariectomized Rat Model

**DOI:** 10.1002/jbm4.10237

**Published:** 2019-11-09

**Authors:** Zeeshan Sheikh, Gang Chen, Faik Al‐Jaf, Marion Thévenin, Kate Banks, Michael Glogauer, Robert N Young, Marc D Grynpas

**Affiliations:** ^1^ Lunenfeld‐Tanenbaum Research Institute Mount Sinai Hospital Toronto Ontario Canada; ^2^ Department of Laboratory Medicine and Pathology University of Toronto Toronto Ontario Canada; ^3^ Faculty of Dentistry University of Toronto Toronto Ontario Canada; ^4^ Faculty of Dentistry Dalhousie University Halifax Nova Scotia Canada; ^5^ Department of Chemistry Simon Fraser University Burnaby British Columbia Canada; ^6^ Division of Comparative Medicine University of Toronto Toronto Ontario Canada; ^7^ Department of Physiology University of Toronto Toronto Ontario Canada; ^8^ Department of Dental Oncology and Maxillofacial Prosthetics Princess Margaret Cancer Centre Toronto Ontario Canada; ^9^ Institute of Biomaterials and Biomedical Engineering University of Toronto Toronto Ontario Canada

**Keywords:** ANABOLICS, BIOMECHANICS, BONE HISTOMORPHOMETRY, OSTEOPOROSIS, PRECLINICAL STUDIES

## Abstract

Pathological bone loss is a regular feature of postmenopausal osteoporosis, and the microstructural changes along with the bone loss make the individual prone to getting hip, spine, and wrist fractures. We have developed a new conjugate drug named C3, which has a synthetic, stable EP4 agonist (EP4a) covalently linked to an inactive alendronate (ALN) that binds to bone and allows physiological remodeling. After losing bone for 12 weeks, seven groups of rats were treated for 8 weeks via tail‐vein injection. The groups were: C3 conjugate at low and high doses, vehicle‐treated ovariectomy (OVX) and sham, C1 (a similar conjugate, but with active ALN at high dose), inactive ALN alone, and a mixture of unconjugated ALN and EP4a to evaluate the conjugation effects. Bone turnover was determined by dynamic and static histomorphometry; μCT was employed to determine bone microarchitecture; and bone mechanical properties were evaluated via biomechanical testing. Treatment with C3 significantly increased trabecular bone volume and vertebral BMD versus OVX controls. There was also significant improvement in the vertebral load‐bearing abilities and stimulation of bone formation in femurs after C3 treatment. This preclinical research revealed that C3 resulted in significant anabolic effects on trabecular bone, and EP4a and ALN conjugation components are vital to conjugate anabolic efficacy. A combined therapy using an EP4 selective agonist anabolic agent linked to an inactive ALN is presented here that produces significant anabolic effects, allows bone remodeling, and has the potential for treating postmenopausal osteoporosis or other diseases where bone strengthening would be beneficial. © 2019 The Authors. *JBMR Plus* published by Wiley Periodicals, Inc. on behalf of American Society for Bone and Mineral Research.

## Introduction

Postmenopausal osteoporosis results in pathological bone loss owing to the negative bone balance caused by increased bone tissue turnover and resorptive activity, which is much greater than the natural bone formation.^1^, ^2^ Cancellous bone has a porous structure that exposes a greater surface area for osteoclast‐based resorption and hence is affected more than cortical bone in osteoporosis.^3^ The microstructural changes along with the bone loss associated with the disease make the individual prone to getting hip, spine, and wrist fractures.^4^, ^5^ Current treatments for postmenopausal osteoporosis are commonly based around using bisphosphonate (BP) drugs and parathyroid hormone (PTH) therapy.^6^, ^7^, ^8^, ^9^

BPs bind selectively to minerals in bone and induce osteoclast apoptosis (via mevalonate pathway interference^8^, ^9^, ^10^), resulting in decreased bone resorption.^8^, ^11^ One of the commonly used BPs is alendronate (ALN), which has demonstrated the ability to decrease spinal fracture risk and result in BMD increase in postmenopausal women.^12^, ^13^ However, long‐term BP use suppresses bone formation and results in a reduction in overall turnover because of the coupling between resorptive and formative processes.^14^, ^15^ PTH has been shown to produce net anabolic effects in humans and rats,^6^, ^7^, ^16^ and teriparatide (Forteo; Eli Lilly, Indianapolis, IN, USA), a recombinant form of PTH, is the only anabolic therapy for osteoporosis that is clinically approved. However, Forteo must be injected daily, and the use of a high‐dose PTH long‐term has been linked with osteogenic sarcoma production in rats.^17^, ^18^, ^19^, ^20^

Prostaglandin E_2_ (PGE_2_) is a natural hormone derived from arachidonic acid and is widely produced within the body that also stimulates bone remodeling.^21^ Studies have shown that PGE_2_ can increase bone formation by stimulating osteoblast differentiation^22^, ^23^, ^24^; it also stimulates osteoclastogenesis and bone‐resorptive capacity in vitro.^25^, ^26^ In vivo, it can produce net anabolic effects with intermittent administration, such as increasing rat cortical bone mass,^27^, ^28^ enhancing rat trabecular bone volume,^29^, ^30^ inducing endosteal and periosteal bone formation,^31^, ^32^ preventing cancellous bone loss caused by ovariectomy,^33^ and improving mechanical strength.^34^ However, PGE_2_ when administered systematically has been shown to produce several adverse effects such as gastrointestinal problems, headache, lethargy, and uterine contractions in humans.^35^ Also, PGE_2_ requires a high dose to achieve strong anabolic bone effects in rats^36^, ^37^ and daily administration.

Research has shown that PGE_2_ affects bone formation and resorption mainly facilitated by the EP4 receptor, which is G protein coupled and increases intracellular cAMP by activating adenylate cyclase.^38^ There is abundant expression of EP4 receptors in primary human osteoblasts and osteoclasts,^39^, ^40^ osteoblastic cell lines in mice and rat bone tissue.^41^ Studies using mice with PGE_2_ receptor knocked‐out have revealed that the EP4 receptor is largely responsible for PGE_2_'s stimulatory effects on bone tissue formation and resorption.^36^, ^42^ EP4 agonists (eg, EP4a) mimic PGE2 effects on bone, such as inducing woven bone formation in mice,^43^ trabecular and cortical bone mass restoration and improving biomechanics in ovariectomized rats,^44^ and stimulation of murine bone resorption.^42^ It has also been demonstrated that PGE2 effects in vivo and in vitro are suppressed when a selective EP4 receptor antagonist is used.^45^, ^46^, ^47^ However, the systemic administration of EP4 agonists results in a series of side‐effects similar to those of native PGE2,^(43)^ which limits their clinical usefulness in osteoporosis treatment despite the overall anabolic effects.

**Figure 1:**
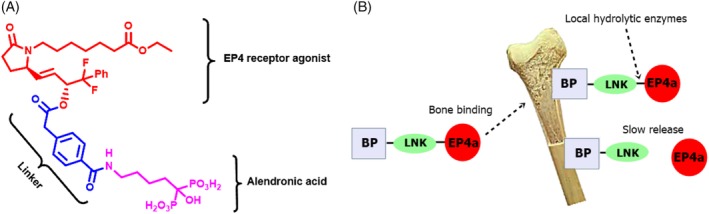
Novel BP‐LK‐EP4a (C3) conjugate drug. (*A*) Chemical structure of the C3 conjugate. (*B*) Schematic depiction of the C3 conjugate drug binding to bone and the slow release of the EP4a via the action of local hydrolytic enzymes.

The diagnosis of osteoporosis is often made after a significant bone tissue loss has already taken place. Conjugate prodrugs such as C1 that liberate an active BP as well as an EP4 agonist, do not allow for bone remodeling to take place because of the inhibition of bone resorption leading to overproduction of bone and partial occlusion of the marrow cavity with endocortical bone.^48^, ^49^, ^50^ To overcome this deficit and to reduce the EP4a associated unwanted side‐effects, we have used a modified conjugate bone‐targeting approach where a synthetic, stable EP4 agonist (EP4a) is covalently linked to an inactive form of alendronate (iALN; shown as BP‐LNK in Fig. 1 *A*) to form a novel bone‐targeting conjugate prodrug C3.^51^, ^52^ The rationale is that after systemic administration, the conjugates should selectively bind to bone surface through iALN's bone targeting and binding ability,^8^ and local hydrolytic enzymes slowly liberate the EP4a component to exert anabolic effects on bone tissue in a sustained release manner (Fig. 1 *B*), while the iALN would be inactive (no antiresorptive effect), would remain attached to bone, and the BP‐LNK bond would be stable and would not liberate active alendronate, thus allowing bone to naturally remodel.

We employed an ovariectomized (OVX) rat model of postmenopausal bone loss with the aim to study the anabolic effects of the novel C3 conjugate on cancellous and cortical bone tissue. Bone loss resulting from OVX in rats shares similar characteristics with postmenopausal bone loss in humans,^53^ which makes it a suitable animal model to use. We have already shown the effects of the C1 conjugate on the lumbar vertebrae and femurs of OVX rats.^48^, ^49^ However, the C1 drug does not allow physiological remodeling and hence is not an ideal therapy for postmenopausal osteoporosis. Our study aims to investigate the in vivo effects of C3 conjugate on bone in a curative 8‐week drug treatment study for OVX‐induced bone loss. We hypothesize that C3 conjugate treatment would promote bone formation and improve bone quality, and in addition allow for physiological tissue‐level remodeling. The effects of the novel conjugated drug C3 on microarchitecture, volumetric bone mineral density (vBMD), and mechanical properties are studied.

## Materials and Methods

### Animals

The University of Toronto Animal Care Committee approved all procedures (Protocol number: 20010975). Charles River Laboratories (Quebec, Canada) provided 70, 3‐month‐old female virgin Sprague–Dawley rats. Sixty rats underwent bilateral ovariectomy (OVX) at Charles River, whereas 10 rats were sham‐operated. The OVX animals lost bone for 12 weeks to establish osteopenia.^54^ The Division of Comparative Medicine (DCM) at the University of Toronto was responsible for housing the animals (two per cage) for the study duration. Light and dark cycles of 12 hours were provided along with free access to reverse osmosis, charcoal‐filtered‐ and ultraviolet‐light‐sterilized H_2_O, and a standard diet for rodents (Tecklad Global 2018; Harlan Laboratories, Indianapolis, IN, USA).

### Treatment

After the animals had lost bone for 12 weeks after OVX, treatment was started. There were seven groups of 10 animals each. Animals were randomized into six groups (OVX animals): The vehicle treatment group had the sham‐operated rats as shown in Table 1. Groups consisted of C3 conjugate at high and low doses (C3H and C3L, respectively), negative control vehicle treated group (OVX) and C1 drug group, inactive alendronate (iALN) used alone to assess the anticipated lack of bisphosphonate antiresorptive activity, and an unconjugated mixture of EP4a and iALN to assess conjugation effects. Intravenous (i.v.) dosing of all groups was conducted via the tail vein (1 mL/kg) once a week for 8 weeks. The investigators were blinded, and the injections and handling were performed by animal facility staff. After the treatment phase was over, the rats were euthanized by cardiac puncture under anesthesia using isoflurane. All drugs and agents administered were provided by DCM and retrieved bone samples were cleaned and treated as per requirement for each anticipated experiment.

**Table 1:** Treatment Groups of the Study

Group	Number	Animal type	Treatment	Dosage (mg/kg)	Molar dosage (μmol/kg)	Frequency	Total dosage (mg/kg)
1. Sham control	10	Sham	Vehicle (PBS)	‐	‐	Weekly	‐
2. OVX control	10	OVX	Vehicle (PBS)	‐	‐	Weekly	‐
3. C3 High dose	10	OVX	C3	5.0	5.8	Weekly	46.4
4. C3 Low dose	10	OVX	C3	2.5	2.9	Weekly	23.2
5. OVX and + ve ctrl	10	OVX	C1	5.0	5.7	Weekly	45.6
6. Inactive ALN	10	OVX	iALN	2.5	5.5	Weekly	44.0
7. Unconjugated mix	10	OVX	iALN + EP4a	2.5 + 2.5	5.5 + 6.0	Weekly	44 + 48

Sham and OVX are healthy and negative controls, C3 groups at both high and low dose, C1 is the OVX plus positive control, inactive alendronate (iALN) is used alone to assess the blockage of bisphosphonate activity and unconjugated mix (iALN + EP4a). All solutions were administered at 1 mL/kg via IV tail‐vein injections.

### Micro‐computed tomography

Postanimal euthanasia, left femurs and sixth lumbar vertebrae were scanned using the Skyscan 1174 Compact Desktop Micro‐CT (Bruker‐MicroCT, Kontich, Belgium) to determine vBMD and bone microarchitecture based on the set‐up described previously.^48^, ^49^ Care was taken to ensure the vertical alignment of the femoral distal region, the femoral shaft, and vertebral body; imaging for all samples was performed using 11.6‐μm^3^ isotropic voxels, 50‐kVp source voltage, 800 μA current, and 3600‐ms exposure time with two frame averages. Images were reconstructed using the SkyScan NRecon software and analyzed using the SkyScan CT‐Analyzer software (version 1.5.0). Analyses were performed using the CTAn software. The vertebral region of interest was defined as the secondary spongiosa of the trabecular compartment, excluding the primary spongiosa near the cranial and caudal vertebral growth plates. The growth plate in the distal femoral bones was used as a reference for image analysis; a region of interest was selected and delineated into 2‐mm segments along the longitudinal direction. The femoral region of interest was defined as a 1‐mm‐thick volume in the middiaphysis. Hydroxyapatite phantoms of 750 and 1300 mg HA/cm^3^ were scanned each day for calibration purposes. Greyscale images were thresholded from 80 to 255, ensuring all bone with density 0.73 g/cm^3^ and above were included in subsequent analyses.

### Histomorphometry

Undecalcified: At 10 and 2 days prior to euthanasia, animals were injected with calcein green (Sigma Aldrich, St. Louis, MO, USA; 10 mg/kg i.p.). Following sacrifice, the left tibias were fixed in 70% ethanol for 5 days, defatted in acetone, and embedded undecalcified in Spurr plastic resin. All static and dynamic histomorphometric analyses were performed using the Bioquant Osteo 11.2.6 MIR software (Bioquant Image Analysis Corporation, Nashville, TN, USA). Osteoid formation was assessed via Goldner's trichrome staining of 5‐mm‐thick coronal sections (Supplementary Fig. SS1).^55^ Bone formation kinetics were measured by fluorescence microscopy using 7‐mm‐thick unstained coronal sections (Supplementary Fig. SS2).

Decalcified: To measure resorption, right tibias were fixed in 10% neutral buffered formalin (NBF) for 5 days, decalcified in 0.5M ethylenediaminetetraacetic acid (EDTA) (pH 7.4), and embedded in bone‐specific paraffin. Tartrate‐resistant acid phosphatase (TRAP) staining was performed on 5‐mm‐thick coronal sections using a standard acid phosphatase leukocyte kit (Sigma‐Aldrich) to measure osteoclastic parameters (Oc.S/BS percent osteoclast surface and N.Oc/BS osteoclast density). The region of interest was defined as the tibial proximal metaphysis beginning 1 mm from the distal end of the growth plate and extending 2 mm into the metaphysis. Histomorphometric parameters were measured following the guidelines of the American Society of Bone and Mineral Research for Bone Histomorphometry.^56^

### Whole‐bone biomechanics

Biomechanical tests were conducted by using an Instron 4465 testing machine (Instron, Canton, MA, USA) based on previous set‐up.^48^, ^49^ Sixth lumbar vertebrae and left femurs were wrapped in saline‐soaked gauze and stored at −20°C. Samples were thawed to room temperature and rehydrated in saline prior to biomechanical testing. Each sixth lumbar vertebra was trimmed to remove intervertebral soft tissue and cartilage; the caudal end was affixed vertically to a metal plate using superglue; and the cranial end was flattened by the addition of polymethylmethacrylate (PMMA; Patterson Dental, St. Paul, MN, USA) to perform vertebral compression testing. Each vertebra was preloaded with 1 N force, and the crosshead was lowered at a rate of 1 mm/min until specimen failure. Force and displacement data were recorded for both tests using the LabVIEW software (National Instruments Corp., Austin, TX, USA) at 2‐Hz sampling frequency.

Three‐point bending tests were performed by using a span of 15 mm and the femurs positioned across a custom‐built apparatus with the posterior side facing downward. Each femur was preloaded with 1 N, and the crosshead was lowered at 0.5 mm/min until specimen failure. All time points and load data were recorded with LabView Acquisition software (LabView v5.0; National Instruments, Austin, TX, USA). Force‐displacement curves were generated from the data collected and normalized to the geometry of the specimen to construct stress–strain curves.

### Back‐scattered electron microscopy

Left tibias were embedded in Spurr plastic and polished to 1‐mm diamond finish using a Phoenix BETA Grinder/Polisher (Buehler, Lake Bluff, IL, USA), and Fimo polymer clay (Fimo Classic, Nuernberg, Germany) was used to mount the blocks on an acrylic glass plate with the bone surface level and facing upward. Blocks were imaged using back‐scattered electron microscopy (BSE; Philips XL300ESEM system, FEI Company, Hillsboro, OR, USA) after carbon coating. Settings were ×80 magnification using of 20 kV at a working distance of 15 mm.

### Statistical analysis

SPSS statistical software (version 21; IBM, Armonk, NY, USA) was utilized to perform the statistical analysis. The results were compared using ANOVA, with Bonferroni correction for post hoc multiple comparison testing. Significance was defined as *p* < 0.05 for two‐tailed probability at 95% CI and data presented as mean ± SD.

## Results

### Treatment effects on the health of animals

The weight of the animals at the OVX/sham surgeries, and the start and end of the 8‐week treatment period were noted and monitored. It was observed that the weights of animals in all OVX groups increased relative to sham at start and end of the study (data not shown). This observation is consistent with weight gain related to OVX as reported previously.^57^ Animal reactions during treatment and general health were observed and no adverse reactions were observed in the OVX and sham groups that were vehicle treated. Two animals in the C1 group suffered from delayed healing at the site (tail vein) of injection. Among other groups, animals in the iALN + EP4a (unconjugated mixture) showed adverse side‐effects, such as diarrhea and hyperventilation, but recovered within 24 to 36 hours. There were no side‐effect reactions observed in animals in the C3H, C3L, and iALN groups.

### The effects of conjugates on bone turnover

**Figure 2:**
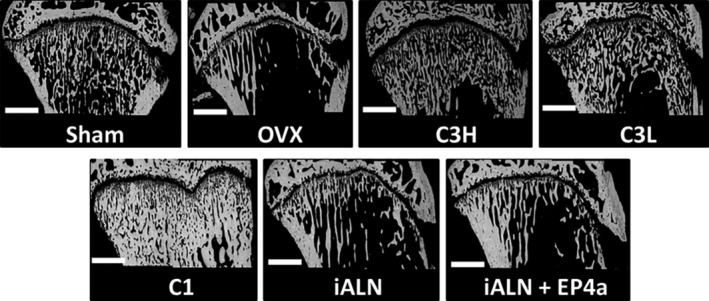
Treatment effects in the proximal tibial metaphysis. Representative images of proximal tibia scanned using BSE (×80 magnification). The scale bar represents 2000 μm.

The treatment effects on the proximal tibial metaphysis produced by administration of various conjugates and solutions are shown in Fig. 2. OVX‐related bone tissue loss was confirmed by reduced bone volume observed in the OVX group relative to the sham group, and C3H, C3L, and C1 conjugate treatments resulted in trabecular bone volume being relative to the OVX group: This observation was dose‐dependent. The C3H and C3L appeared to have bone volume measured somewhat similar to the original sham levels. However, the C1 conjugate treatment exhibited excess bone volume. The iALN and the iALN + EP4a treatments did not result in restoration of bone volume; they appeared very similar to OVX (Fig. 2).

Histomorphometric bone turnover results are presented in Table 2; it can be seen that EP4a and ALN conjugation is critical to the anabolic efficacy of the conjugate drugs. Elevated tissue‐level bone turnover caused by ovariectomy was evidenced by the dynamic bone parameters being significantly increased, including the mineral apposition rate (MAR), mineralizing surface (MS/BS), and the rate of bone formation (BFR/BS); also, the percentage osteoclast surface and percentage osteoid volume were more in the OVX group when compared with the sham group (Table 2).

**Table 2:** Histomorphometric Analysis of Bone Turnover in the Proximal Tibial Metaphysis at Tissue Level

Analysis type	Sham mean ± SD	OVX mean ± SD	C3H mean ± SD	C3L mean ± SD	C1 mean ± SD	iALN mean ± SD	iALN + Ep4a mean ± SD
BV/TV (%)	56.17 ± 4.15	25.81 ± 5.74*	57.95 ± 3.84$	51.63 ± 8.08$	65.00 ± 5.01* ^,^ $ ^,^ &	27.14 ± 2.73* ^,^ # ^,^ & ^,^ Δ	26.82 ± 3.63* ^,^ # ^,^ & ^,^ Δ
MS/BS (%)	6.38 ± 1.21	18.09 ± 4.38*	32.27 ± 4.04* ^,^ $	27.14 ± 5.23* ^,^ $	34.50 ± 9.51* ^,^ $ ^,^ # ^,^ &	16.82 ± 5.09* ^,^ & ^,^ # ^,^ Δ	17.49 ± 6.18* ^,^ & ^,^ # ^,^ Δ
MAR (μm/day)	0.85 ± 0.13	1.35 ± 0.07*	2.12 ± 0.51*	1.84 ± 0.47*	2.24 ± 0.36* ^,^ $ ^,^ # ^,^ &	1.34 ± 0.06* ^,^ Δ	1.35 ± 0.03* ^,^ Δ
BFR/BV(μm/day/mm^2^)	1.36 ± 0.11	6.82 ± 1.26*	4.26 ± 1.24* ^,^ $	3.61 ± 1.03* ^,^ $ ^,^ #	4.90 ± 1.90* ^,^ $ ^,^ # ^,^ &	6.59 ± 1.00* ^,^ # ^,^ & ^,^ Δ	6.67 ± 1.43* ^,^ # ^,^ & ^,^ Δ
BFR/BS (μm/day/mm)	0.072 ± 0.01	0.21 ± 0.052*	0.43 ± 0.08* ^,^ $	0.37 ± 0.12* ^,^ $	0.48 ± 0.11* ^,^ $ ^,^ # ^,^ &	0.23 ± 0.07* ^,^ # ^,^ &	0.28 ± 0.09* ^,^ # ^,^ &
OV/BV (%)	0.034 ± 0.001	0.20 ± 0.037*	0.042 ± 0.005$	0.035 ± 0.002$	0.077 ± 0.01* ^,^ $	0.19 ± 0.012* ^,^ # ^,^ & ^,^ Δ	0.22 ± 0.025* ^,^ # ^,^ & ^,^ Δ
OS/BS (%)	0.511 ± 0.043	1.57 ± 0.344*	0.724 ± 0.07$	0.91 ± 0.024$	0.92 ± 0.16* ^,^ $	1.822 ± 0.32* ^,^ # ^,^ & ^,^ Δ	1.79 ± 0.553* ^,^ # ^,^ & ^,^ Δ
Oc.S/BS (%)	4.01 ± 1.01	9.30 ± 1.06*	9.01 ± 1.02*	8.92 ± 1.18*	4.41 ± 1.91$ ^,^ # ^,^ &	8.32 ± 1.30* ^,^ Δ	9.04 ± 1.42* ^,^ Δ
N.Oc/BS (1/mm)	2.44 ± 0.51	3.91 ± 0.38*	3.53 ± 0.30*	3.60 ± 0.31*	1.50 ± 0.51* ^,^ $ ^,^ # ^,^ &	3.81 ± 0.31* ^,^ Δ	3.78 ± 0.22* ^,^ Δ

BV/TV = trabecular bone volume; MS/BS = mineralizing surface; MAR = mineral apposition rate; BFR/BV = bone formation rate normalized over bone volume; BFR/BS = bone formation rate (surface referent); OV/BV = percent osteoid volume; OS/BS = percent osteoid surface, Oc.S/BS = percent osteoclast surface; N.Oc/BS = osteoclast density.

* *p* < 0.05, compared with SV;

$ *p* < 0.05, compared with OVX;

# *p* < 0.05, compared with C3H;

& *p* < 0.05, compared with C3L;

Δ *p* < 0.05, compared with C1.

Treatment with conjugate drugs resulted in stimulation of bone formation kinetics, as C3H and C3L were increased compared with all other groups (apart from C1 which was even greater) in MAR, MS/BS, and BFR/BS. Relative to OVX, C3H and C3L led to 78% and 50% increase in MS/BS, 57% and 36% increase in MAR, and 104% and 76% increase in BFR/BS, respectively. However, these parameters did not increase in the iALN and iALN+EP4a groups, indicating that EP4a and ALN conjugation was very critical to the anabolic efficacy. Treatment with C1 resulted in significant suppression of osteoclast density (N.Oc/BS) and percent osteoclast surface (Oc.S/BS; NS) when compared with OVX, indicating an inhibitory effect on resorption. However, this significant suppression was not observed in the C3H and C3L groups, which points to the inactivity of the ALN. The Oc.S/BS and N.Oc/BS were decreased in the C1‐treated group in comparison to OVX, which indicated bone resorption being suppressed at the tissue level. This decrease was not observed for the C3H‐ and C3L‐treated groups.

### The effect of conjugates on trabecular microarchitecture and mechanical properties

**Figure 3:**
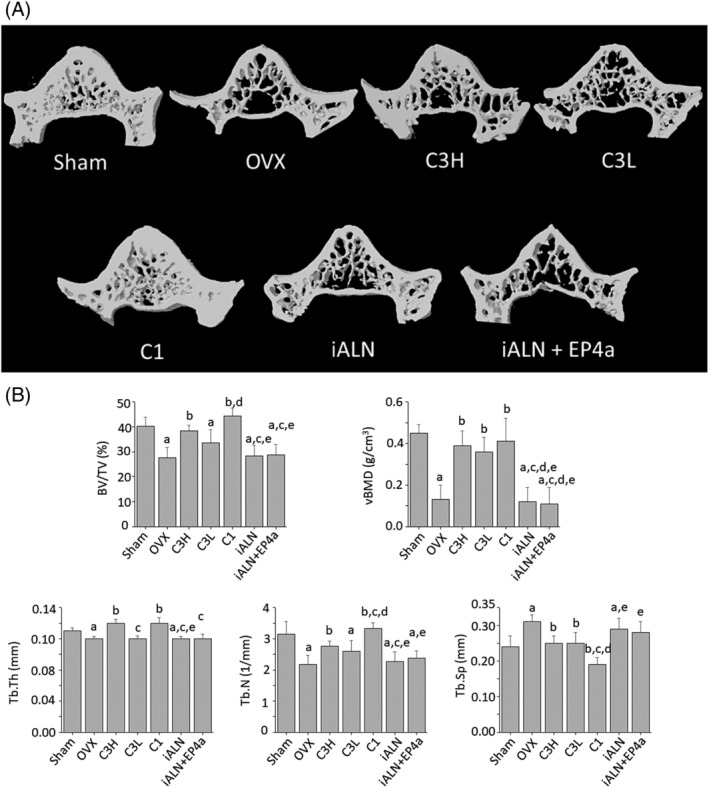
Treatment effects on the microarchitecture of the sixth lumbar vertebrae. (*A*) Representative μCT cross‐sectional 3D images of the L6 vertebrae. The scale bar represents 2000 μm. (*B*) L6 vertebra trabecular bone structural indices measured by μCT analysis. BV/TV = percent bone volume; vBMD = volumetric bone mineral density; Tb.Th = trabecular thickness; Tb.N = trabecular number; Tb.Sp = trabecular separation. a*p* < 0.05, compared with sham; b*p* < 0.05, compared with OVX; c*p* < 0.05, compared with C3H; d*p* < 0.05, compared with C3L; e*p* < 0.05, compared with C1. Mean ± SD.

**Figure 4:**
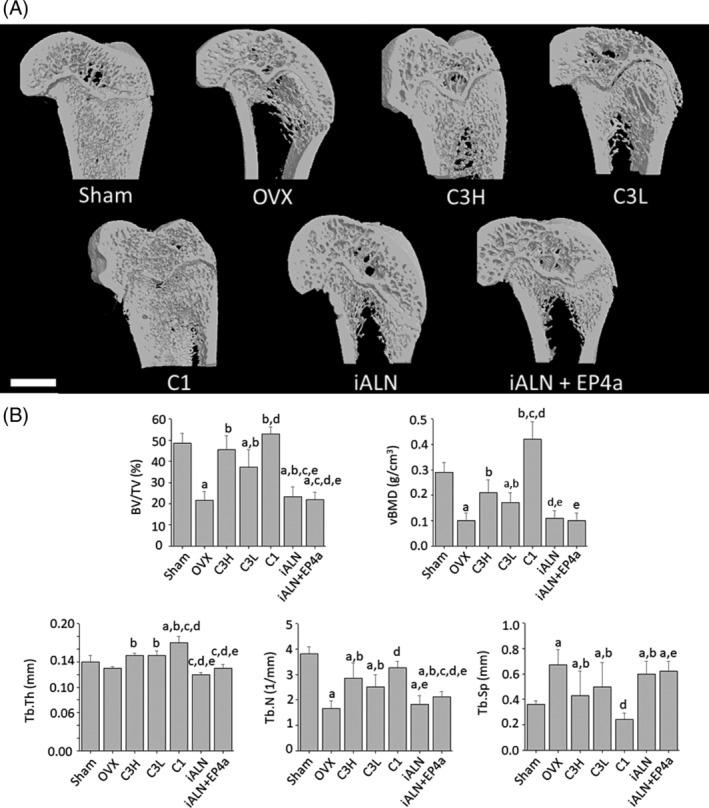
(*A*) Representative μCT sagittal 3D images of distal left femora. The scale bar represents 2000 μm. (*B*) Femur trabecular bone structural indices measured by μCT analysis. BV/TV = percent bone volume; vBMD = volumetric bone mineral density; Tb.Th = trabecular thickness; Tb.N = trabecular number; Tb.Sp = trabecular separation. a*p* < 0.05, compared with sham; b*p* < 0.05, compared with OVX; c*p* < 0.05, compared with C3H; d*p* < 0.05, compared with C3L; e*p* < 0.05, compared with C1. Mean ± SD.

The representative μCT cross‐sectional images demonstrated the visible differences between the L6 vertebras from all seven groups analyzed (Fig. 3 *A*). Structural indices upon examination of the vertebral microarchitecture confirmed bone loss caused by OVX by reduced percent bone volume (BV/TV) and trabecular number (Tb.N) in the OVX group compared with the sham group, along with increase in trabecular separation (Tb.Sp) and a reduction in trabecular thickness (Tb.Th) and tissue‐level bone density (vBMD; Fig. 3 *B*). In the OVX group, the treatment with C3H and C1 increased BV/TV by increasing Tb.N and the trabecular thickness (Tb.Th), which decreased Tb.Sp and improved the vBMD significantly. Dose‐dependent effects were noted for these parameters between C3H and C3L groups and the change in BV/TV did not reach significance for C3L relative to OVX. Although C1 reached sham levels for vBMD, it exceeded sham levels for BV/TV. The groups treated with iALN and iALN + EP4 were very similar to the OVX groups in terms of all indices measured (Fig. 3 *B*). The representative μCT images demonstrated the visible differences between the left femurs from all seven groups analyzed (Fig. 4 *A*). The femur trabecular bone structural indices measured by μCT revealed similar results with one exception: Treatment with C1 surpassed sham levels for both vBMD and BV/TV (Fig. 4 *B*).

**Figure 5:**
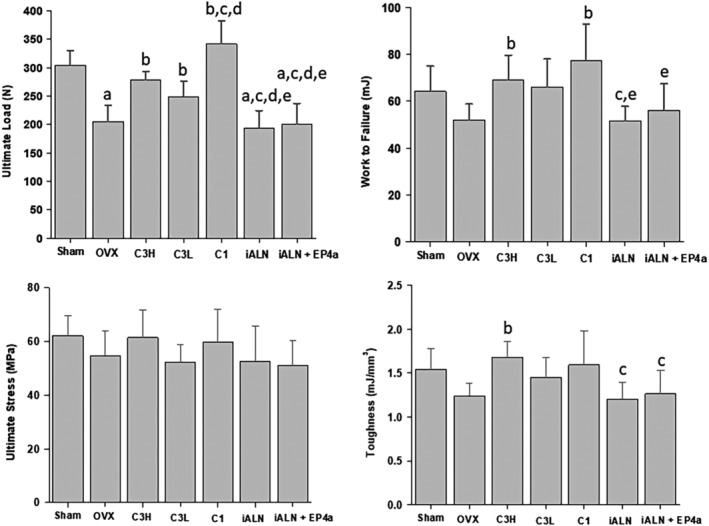
Mechanical properties of the L6 vertebrae determined by vertebral compression testing. a*p* < 0.05, compared with sham; b*p* < 0.05, compared with OVX; c*p* < 0.05, compared with C3H; d*p* < 0.05, compared with C3L; e*p* < 0.05, compared with C1. Mean ± SD.

Compression testing of the vertebrae revealed that treatment with C3H, C3L, and C1 resulted in a dose‐dependent increase in ultimate load relative to OVX, suggesting improvement in vertebral strength (Fig. 5). Work to failure also increased relative to OVX in C3H and C1 treatments, but not for C3L. This suggested that the ability to absorb energy also improved for samples treated with C3H and C1. It was noted that both ultimate load and work to failure were beyond sham levels for the C1 group. The ultimate stress was not changed across all treatment groups after normalizing for sample geometry. The same was observed for toughness apart from in the C3H group where it was greater than OVX (Fig. 5).

### Conjugate effects on cortical bone and the mechanical properties

**Figure 6:**
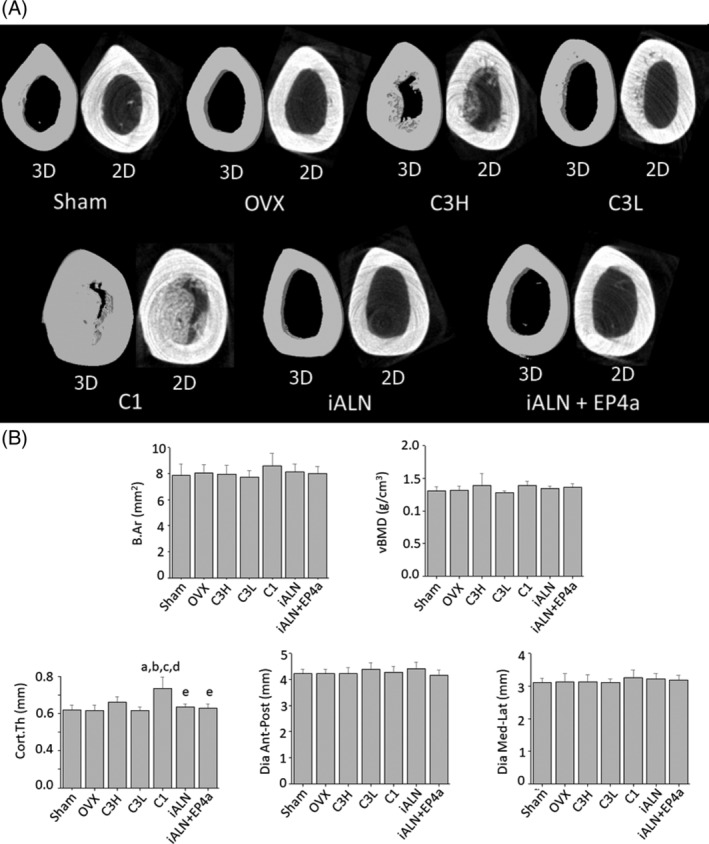
Treatment effects in cortical bone. (*A*) Representative cross‐sectional μCT 3D and 2D images of the left femora at middiaphysis. The scale bar represents 3000 μm (*B*) femur cortical bone structural indices measured by μCT analysis. B.ar = cross‐sectional bone area; vBMD = volumetric bone mineral density; Cort.Th = cortical thickness; Dia ant‐post = diameter anterior to posterior dimension; Dia med‐Lat = diameter medial to lateral dimension. a*p* < 0.05, compared with sham; b*p* < 0.05, compared with OVX; c*p* < 0.05, compared with C3H; d*p* < 0.05, compared with C3L; e*p* < 0.05, compared with C1. Mean ± SD.

**Figure 7:**
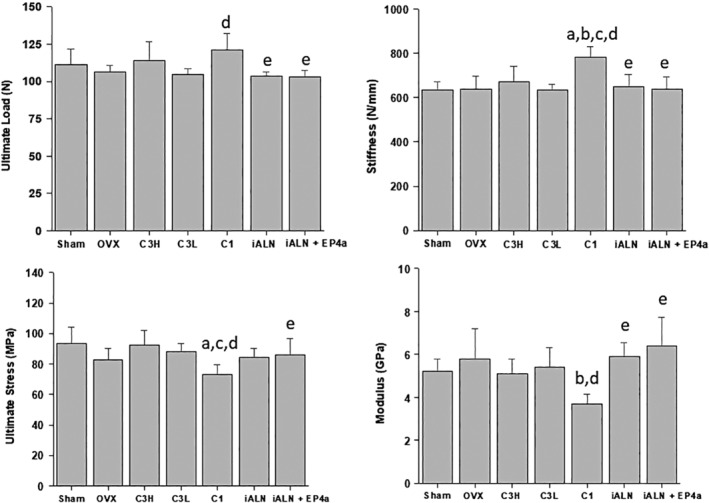
Mechanical properties of the femur determined by three‐point bending tests. a*p* < 0.05, compared with sham; b*p* < 0.05, compared with OVX; c*p* < 0.05, compared with C3H; d*p* < 0.05, compared with C3L; e*p* < 0.05, compared with C1. Mean ± SD.

Upon μCT analysis of femoral midshaft cortical bone, it was revealed that C1 conjugate produced near‐occlusion of the marrow cavity by endocortical woven bone formation (Fig. 6 *A*). This effect was also dose‐dependently seen to a lesser extent with the C3 conjugate treatment. This endocortical bone formation was limited to the cortical portion of the middiaphysis only, and not observed in any other part of the bone samples. Femur cortical bone structural indices measured by μCT analysis showed that the cross‐sectional bone area (B.Ar) and vBMD were unchanged among all groups. However, the cortical thickness significantly increased in the C1 treatment group when compared with all other groups (Fig. 6 *B*). Three‐point bending tests demonstrated that mechanical properties in cortical bone were similar and comparable in all groups except C1, which displayed increased stiffness and ultimate load, but decreased modulus and ultimate stress (Fig. 7).

## Discussion

This research was designed and conducted to evaluate the in vivo effects of the novel C3 conjugate drug, which contains an inactive bisphosphonate (iALN) and an active EP4 receptor agonist (EP4a), in an OVX‐induced osteoporosis model in rats. The C3 was developed as a conjugate to allow local delivery of EP4a's anabolic ability by linking with the bone‐targeting bisphosphonate (iALN). This was done to allow sustained release of EP4a and to eliminate the side‐effects associated with systemic administration of EP4a. Our study design employed a model of postmenopausal osteopenia,^53^ with bone loss of 8 weeks after OVX followed by 8 weeks of treatment once weekly.

Reactions to the drugs being administered to rats were noted, and no adverse reactions were observed in the OVX and sham groups that were vehicle treated. Animals in the iALN + EP4a (unconjugated mixture) showed diarrhea and hyperventilation as reactions (within few minutes of administration). This was observed as i.v. injection results in rapid distribution of the drug solutions via circulation and the lack of conjugation allowed for EP4a to free circulate throughout the circulatory system and produce the unwanted side‐effects. In contrast, the C3H and C3L groups had no adverse reactions, which strongly suggests that the EP4a and ALN‐LK conjugation helped to eliminate the EP4a‐induced systemic side‐effects.

Our results for OVX‐induced bone loss were consistent with other reports,^48^, ^49^, ^58^ such as lumbar vertebral bone loss resulting in vBMD reduction at tissue level and elevated bone turnover in proximal tibial metaphysis. Our results showed that C3 conjugate resulted in the histomorphometric bone formation indices being increased dose‐dependently compared with OVX, indicating promotion of osteoblast recruitment, stimulation of osteoblast activity, and an increase in the rate of bone formation.^28^, ^29^, ^59^ Also, these tissue‐level anabolic effects resulted in de novo bone formation (trabecular) and vertebral mechanical strength improvement, which is consistent with the EP4a‐associated anabolic effects in vivo as reported in the literature.^29^, ^43^, ^60^ Our results showed that treatment with C3H and C3L did not decrease the osteoclast density and percent osteoclast surface compared with the OVX group. This confirmed that the inactive‐BP component of C3 (iALN) did not lead to inhibitory effects on tissue‐level resorption. However, treatment with C1 resulted in decreased osteoclast density and percent osteoclast surface: This can be attributed to the liberated alendronate's well‐established antiresorptive effects.^8^, ^61^

The EP4a and ALN‐LK components of C3 being conjugated were shown to be vital to the drug anabolic activity and efficacy; unlike results from the C3H, C3L, and C1 groups, treatment with iALN + EP4a did not increase MS/BS, MAR, and BFR/BS. The conjugation would be expected to lead to greater and more sustained delivery of EP4a to bone as the iALN component in the C3 conjugate drug is expected to strongly bond to bone and deliver EP4a in close proximity to the bone via enzymatic hydrolysis. Whereas in the iALN + EP4a group, EP4a was distributed via circulation throughout the body and was quickly removed from the bloodstream (half‐life of 1 to 2 hours).^51^ Previous studies have demonstrated the anabolic effects produced by daily administration of EP4 receptor agonists.^43^, ^44^, ^62^ This puts emphasis on the importance of the C3 conjugate treatment that results in the production of significant anabolic effects, even with once‐a‐week administration.

The conjugate treatments resulted in increased woven bone formation as observed by μCT analysis of femoral midshaft cortical bone. C1 administration was shown to cause almost complete occlusion of the marrow cavity and significantly increased the cortical thickness relative to all other groups. This resulted in the C1 group having reduced mechanical properties, as evidenced by a decrease in modulus and ultimate stress. A dose‐dependent increase in woven bone formation was also seen in the C3 conjugate groups, but to a lesser extent when compared with the C1 treatment group. Ultimately, this did not cause a decrease in the mechanical properties. The promotion of endocortical woven bone formation is comparable to the effects of PGE_2_ as found in the literature previously.^22^, ^28^, ^30^, ^49^, ^63^

Unlike the results from C1 therapy, the ideal osteoporosis treatment should result in the restoration of trabecular bone volume without overproduction of bone. It is already known that the administration of high‐dose alendronate (actively antiresorptive) over time results in the oversuppression of bone turnover, compromised bone structure, and decreased mechanical properties.^64^ The inactive alendronate, iALN, used in the C3 conjugate does not block the resorptive ability of osteoclasts; future studies that focus on dose optimization could potentially prevent endocortical bone formation.

In our study, the C3 conjugate treatment produced a strong dose‐dependent trabecular bone volume increase in the lumbar vertebrae, anabolic effects in the proximal tibial metaphysis, and improved vertebral mechanical properties. These effects produced by the administration of the conjugate are extremely pertinent to the management of postmenopausal osteoporosis. A limitation of this study was that the treatment phase was only 8 weeks long and that young (12 weeks old) rats were used, which are not an ideal model for postmenopausal osteoporosis. Also, we could not include a baseline sham group to directly compare the results from the sham and treatment groups after 8 weeks. Future studies are required to evaluate the medium‐ to long‐term effects of the C3 conjugate and to calculate the optimal dose for best clinical results. We report here for the first time a combined therapy, using an anabolic selective EP4 agonist agent and an inactive‐ALN, which demonstrates anabolic effects that reverse osteopenia and allow bone remodeling.

## Disclosures

RNY reports that he is principle in the company, Mesentech Inc. (Vancouver, BC, Canada), who has licensed rights to the active compound used in this study. In addition, RNY has a patent US Provisional Patent Application No. 62/175,118 (June 12, 2015). A PCT (Patent Cooperation Treaty) application was filed June 12, 2016 and was published on December 15, 2016, as WO 2016/199111 A1 pending. GC has patent US2018170951A1 pending. MG and MDG report that they are scientific advisers to Mesentech Inc.

## Supporting information

Supplementary Fig. S1. Representative images of proximal tibia with Goldner's Trichrome staining. The scale bars represent 1000 μm.

Supplementary Fig. S2. Representative images of proximal tibia for dynamic histomorphometry using calcein green. The scale bars represent 1000 μm and 200 μm.
