# Supplementary figures and images for: In Vivo Bone Effects of a Novel Bisphosphonate‐EP4a Conjugate Drug (C3) for Reversing Osteoporotic Bone Loss in an Ovariectomized Rat Model
Source: JBMR Plus. 2019 Nov 9;3(12):e10237. doi: 10.1002/jbm4.10237 (PMC6894726; doi:10.1002/jbm4.10237)

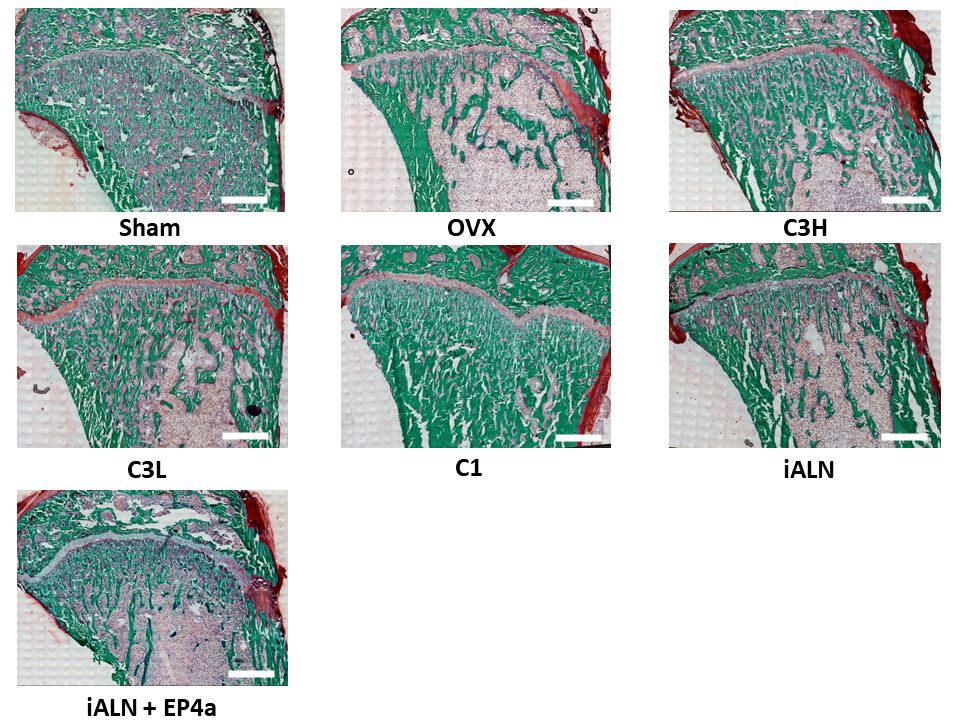

Supplement: Supplementary file 1 — Supplementary Fig. S1. Representative images of proximal tibia with Goldner's Trichrome staining. The scale bars represent 1000 μm. [file JBM4-3-na-s001.tif]

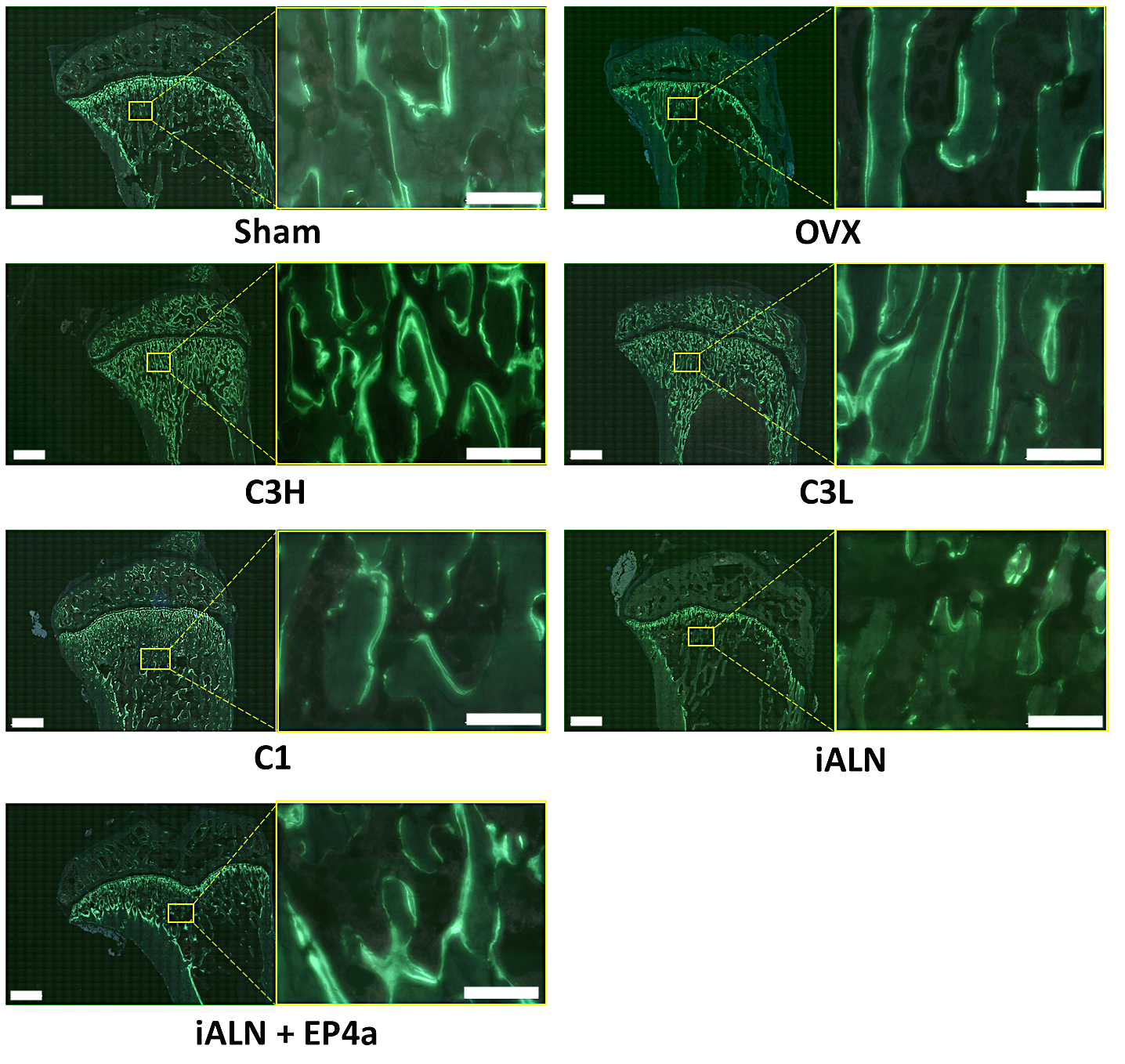

Supplement: Supplementary file 2 — Supplementary Fig. S2. Representative images of proximal tibia for dynamic histomorphometry using calcein green. The scale bars represent 1000 μm and 200 μm. [file JBM4-3-na-s002.tif]
